# ﻿On males of two poorly known *Qianlingula* species from China (Araneae, Pisauridae)

**DOI:** 10.3897/zookeys.1255.144956

**Published:** 2025-10-14

**Authors:** Tian-Yu Ren, Qian-Le Lu, Zhi-Sheng Zhang

**Affiliations:** 1 Key Laboratory of Eco-environments in Three Gorges Reservoir Region (Ministry of Education), School of Life Sciences, Southwest University, Chongqing 400715, China Southwest University Chongqing China; 2 College of Life Sciences and Oceanography, Shenzhen University, Shenzhen 518000, China Shenzhen University Shenzhen China

**Keywords:** Description, first record, morphology, taxonomy

## Abstract

The males of *Qianlingula jiafu* Zhang, Zhu & Song, 2004 and *Q. turbinata* Zhang, Zhu & Song, 2004 are described here for the first time. Detailed descriptions and color photographs of two species are provided, along with a distribution map of all three *Qianlingula* species.

## ﻿Introduction

The spider family Pisauridae Simon, 1980 currently comprises 234 extant species in 44 genera distributed almost globally (WSC 2025). Approximately 100 species have been described based on a single sex (76 from females and 24 from males), including seven species that are known only from females in China.

The genus *Qianlingula* Zhang, Zhu & Song, 2004 comprises three species distributed across the Chinese provinces of Hunan, Guangdong, Guizhou, Hainan, and Fujian. Only both sexes of the type species of this genus are known. While examining pisaurid specimens from southern China, we identified previously unknown males of two other species. The purpose of this paper is to provide their descriptions.

## ﻿Materials and methods

**Figure 1.**
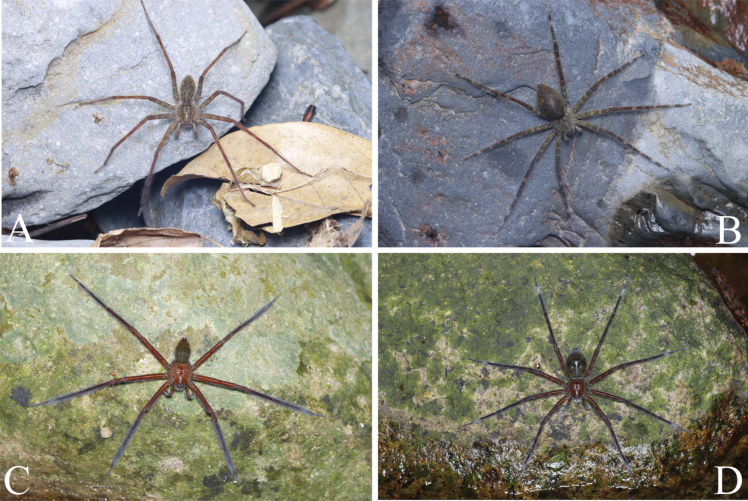
Photos of living specimens. A, B. *Qianlingula jiafu* (A. female, B. male); C, D. *Qianlingula turbinata* (C. Female; D. Male). Photos by Qian-Le Lu.

All specimens are preserved in 75% ethanol and were examined, photographed, and measured using a Leica M205A stereomicroscope equipped with a drawing tube, a Leica DFC450 camera, and LAS software v. 4.6. Male palps and epigynes were examined and illustrated after dissection. Epigynes were cleared by immersing them in pancreatin (Álvarez-Padilla and Hormiga 2007). Photographs of live specimens were taken using a Canon 90D camera with Laowa FF 100 mm F2.8 CA-Dreamer Macro 2× lens (Fig. 1). Eye sizes were measured as the maximum dorsal diameter. Leg measurements are shown as total length (femur, patella + tibia, metatarsus, tarsus). The number of spines is listed for each segment in the following order: dorsal (d), prolateral (p), retrolateral (r), ventral (v) (in femora and patellae ventral spines are absent and fourth digit is omitted in the spination formula) All measurements are given in millimetres. Specimens examined here are deposited in the Museum of Hebei University (**MHBU**, types) and the Collection of Spiders, School of Life Sciences, Southwest University, Chongqing, China (**SWUC**, non-types). Terminology follows Zhang et al. (2004) and Sierwald (1989), except for that of the median plate.

Abbreviations used in the text: **ALE**, anterior lateral eyee; **AME**, anterior median eyee; **Fe**, femure; **Mt**, metatarsuse; **Pa**, patellae; **PLE**, posterior lateral eyee; **PME**, posterior median eyee; **Ti**, tibia.

## ﻿Taxonomy

### ﻿Family Pisauridae Simon, 1980

#### ﻿Genus Qianlingula

Taxon classificationAnimaliaAraneaePisauridae

Zhang, Zhu & Song, 2004

ADD0003C-AA0B-5BEF-9D2B-147EBEAC159C

Qianlingula Zhang, Zhu & Song, 2004: 399.

##### Type species.

*Qianlingula bilamellata* Zhang, Zhu & Song, 2004 from China.

##### Comments.

All three species belonging to the genus were treated in two publications only Zhang et al. (2004) and Yin et al. (2012).

##### Diagnosis.

The genus is similar to *Thalassius* Simon 1885 in body shape and eye pattern, but it can be easily distinguished from the latter by: median plate (MP) of epigyne strongly sclerotized, vs weakly sclerotized, posterior margin of lateral wall (LW) distant from each other, vs near each other; male palp with distinct lamellar retrolateral tibial apophysis (RTA) divided into 2 branches, vs RTA absent; bulb elliptical, with spine-like embolic basic process (EBP), vs EBP absent; embolus (E) running clockwise, entirely filamentous vs short and hooked; tip of fulcrum (Ful) ﬁne, vs thick; conductor (C) crescent-shaped, with two guiding lamellae vs small and lacking guiding lamellae.

##### Description.

See Zhang et al. (2004). Chelicerae brown, with three promarginal and three retromarginal teeth. Embolus complex: with fulcrum (Ful) and basal process (EBP); fulcrum long, curved clockwise as long as embolus; basal process spine-like.

##### Composition.

Total three species in this genus, *Q. bilamellata* (♀♂), *Q. jiafu* Zhang, Zhu & Song, 2004 (♀), *Q. turbinata* Zhang, Zhu & Song, 2004 (♀).

#### ﻿ Qianlingula jiafu

Taxon classificationAnimaliaAraneaePisauridae

CB90ECD1-F364-5B15-B618-860ADF80AD65

**Figure 2.**
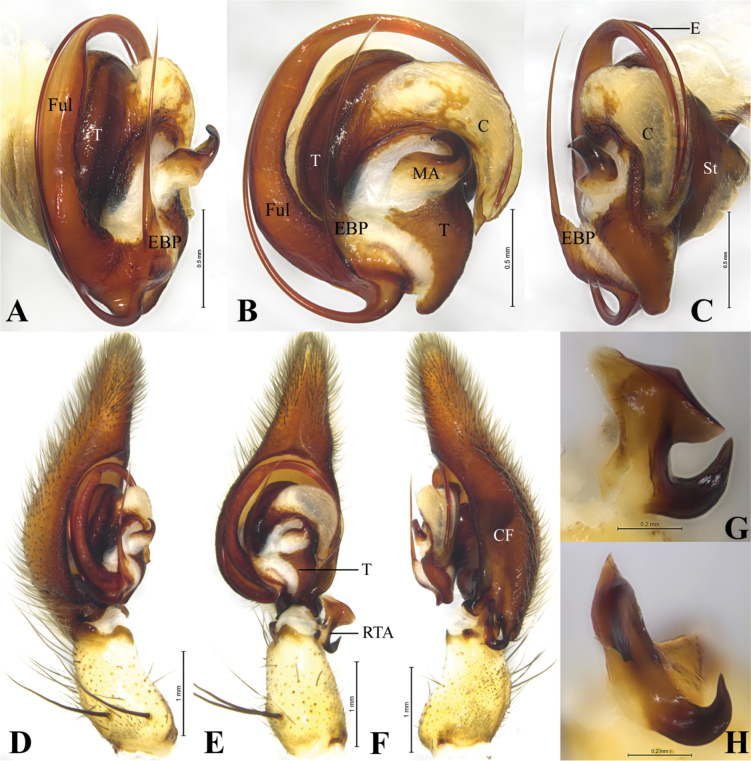
*Qianlingula jiafu*, male. A–C. bulb, prolateral, ventral and retrolateral view; D–F. Palp, rolateral, ventral and retrolateral view; G, H. Retrolateral tibial apophysis of palp, ventral and retrolateral view. Abbreviations: C = conductor, CF = cymbial furrow, E = embolus, EBP = embolic basic process, Ful = fulcrum, MA = median apophysis, RTA = retrolateral tibial apophysis, St = Subtegulum, T = tegulum.

**Figure 3.**
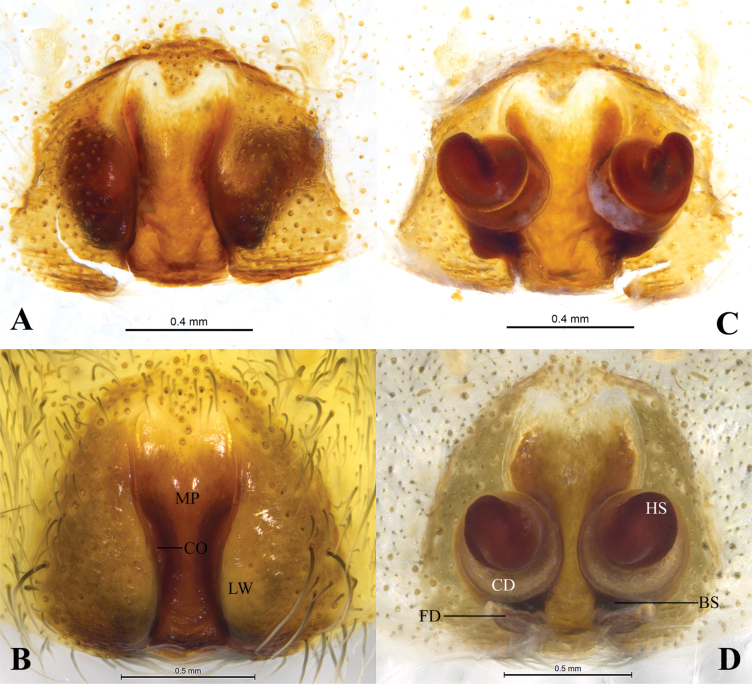
*Qianlingula jiafu*, female (holotype A, C). A, B. Epigyne, ventral view; C, D. Same, dorsal view. Abbreviations: BS = base of spermatheca, CD = copulatory duct, CO = copulatory opening, FD = fertilization duct, HS = head of spermatheca, LW = lateral wall, MP = median plate.

**Figure 4.**
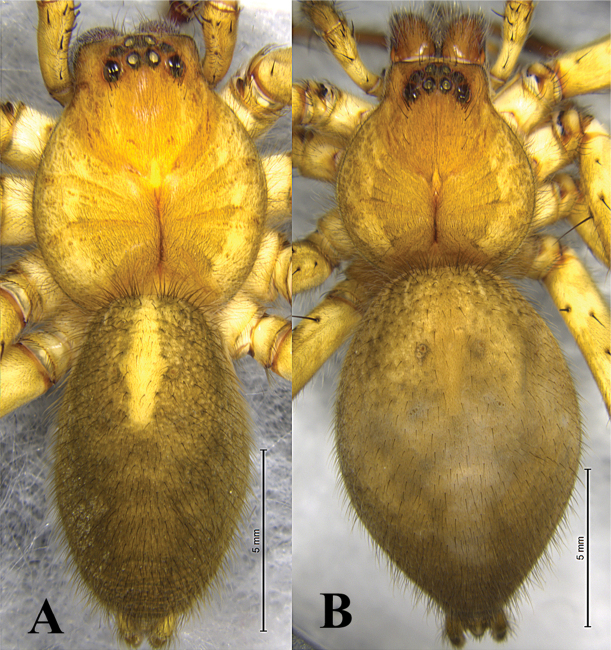
Habitus of *Q. jiafu*. A. Male; B. Female.

Figs 1A, B, 2, 3, 4, 7

Qianlingula jiafu Zhang et al., 2004: 400, figs 156–159 (♀); Yin et al. 2012: 895, fig. 450a–d (♀).

##### Material examined.

***Holotype*** • ♀, China, Hunan, Dayong Co. (Zhangjiajie Ct.), 20.07.1981, J.F. Wang leg. (MHBU, Figs 3A, C). ***Paratypes*** • 2♂ 2♀, China, Guangxi, Longsheng Co., Sanmen Twn., Huaping Vill., Huaping Nature Reserve, 25°37'54"N, 109°54'30"E, elev. 555 m, 30.04.2023, L.Y. Wang et al. leg. (SWUC, Figs 1A, B, 2, 3B, D, 4).

##### Diagnosis.

**Figure 5.**
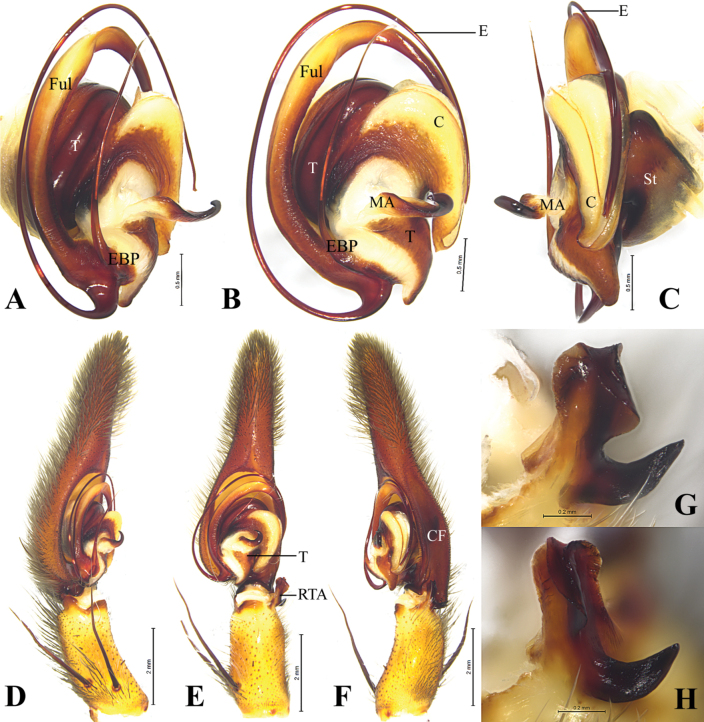
*Qianlingula turbinata*, male. A–C. Bulb, prolateral, ventral and retrolateral view; D–F. Palp, rolateral, ventral and retrolateral view; G, H. Retrolateral tibial apophysis of palp, ventral and retrolateral view. Abbreviations: C = conductor, CF = cymbial furrow, E = embolus, EBP = embolic basic process, Ful = fulcrum, MA = median apophysis, RTA = retrolateral tibial apophysis, St = Subtegulum, T = tegulum.

**Figure 6.**
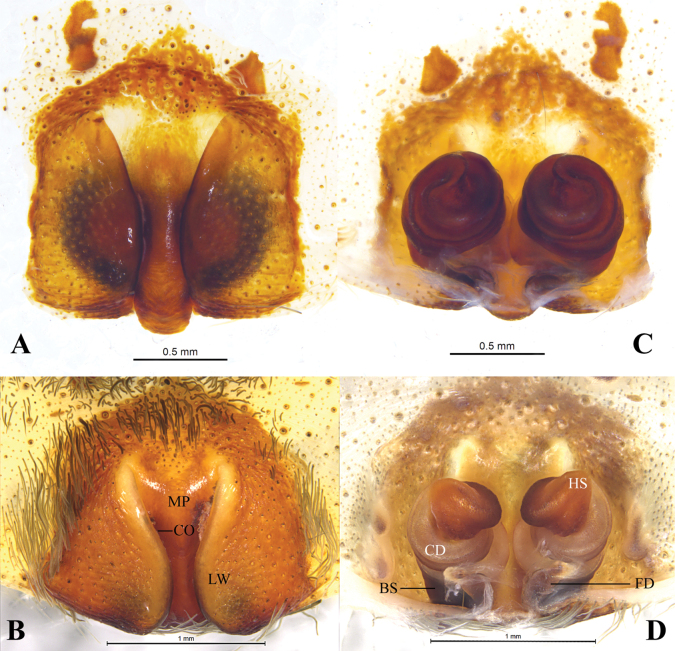
*Qianlingula turbinata*, female (holotype A, C). A, B. Epigyne, ventral view; C, D. Same, dorsal view. Abbreviations: BS = base of spermatheca, CD = copulatory duct, CO = copulatory opening, FD = fertilization duct, HS = head of spermatheca, LW = lateral wall, MP = median plate.

This species resembles *Q. turbinata* (Figs 5, 6) in having a bifurcated retrolateral tibial apophysis (RTA), a crescent-shaped and grooved conductor (C), a long and slender embolus (E) originated at approximately 6-o’clock position, spine-like embolic process (EBP) pointing anteriorly and a slit-like copulatory openings (CO) of epigyne, but differs by smaller body size (16.23–18.14) (Fig. 6B, D, vs 27.45–34.33); relatively thick anterior branch of retrolateral tibial apophysis (Fig. 2E–H, vs with lamellar and pleated in *Q. turbinata*, Fig. 5E–H); relatively pointed tip of conductor in ventral view (Fig. 2B, vs blunt, Fig. 5B). Posterior part of epigynal median plate wider than half of anterior part (Fig. 3A, B, vs narrower than half of the anterior part, Fig. 6 A, B).

##### Description.

**Male** (Figs 1A, 4A). Total length 16.23. Carapace 7.62 long, 6.47 wide; abdomen 9.16 long, 5.33 wide. Carapace yellow-brown, margin dark brown, with red brown radial furrow. Dorsum of abdomen dark brown, cardiac pattern rhomboid yellow-brown, with two pairs of brown muscular mark. Eye sizes and interdistances: AME 0.43, ALE 0.34, PME 0.41, PLE 0.43; AME–AME 0.26, AME–ALE 0.15, PME–PME 0.22, PME–PLE 0.53. Clypeus height 0.55. Spination of left leg I: Fe 3d 5p 5r; Pa 1d 1p 1r; Ti 2d 2p 2r 4-4v; Mt 3d 3p 3r 3-0v. Leg measurements: I 42.75 (10.80, 15.56, 11.98, 4.41); II 45.58 (11.65, 16.83, 12.75, 4.35); III 36.49 (9.88, 12.94, 10.05, 3.62); IV 40.67 (10.15, 14.00, 12.20, 4.32).

***Palp*** (Fig. 2). Tibia ca 2 times longer than wide in retrolateral view, ca 0.4 of femur length, as long as patella, proximal part wider than distal RTA length in ventral view, with two strong prolateral spines; tibial apophysis bifurcated, anterior branch thick, with lamellar margin, posterior branch hook-like, curved, extending dorso-anteriorly, with pointed tip. Cymbium droplet-shaped, 2 times longer than wide, ca 1.7 times of tibia length and width; cymbial furrow (CF), ca 0.6 times of cymbium length; tip 0.4 times of cymbial length; dorso-posterior part extended posteriorly (Fig. 2F). Bulb slightly elliptical, as long as wide; proximal terminal part of tegulum (T) funnel-shaped and curved; subtegulum (St) triangular in retrolateral view; median apophysis (MA) located centrally, hook-like. Conductor crescent-shaped in ventral view, with groove in retrolateral view (holding fulcrum (Ful) and embolus), with pointed tip. Embolus complex: with fulcrum (Ful) and basal process (EBP); fulcrum long, curved clockwise as long as embolus; basal process spine-like. Embolus proper originating at approximately 6-o’clock position, long, running clockwise surrounded tegulum, distal part resting in long groove of fulcrum and terminating at ca 3-o’clock position.

**Female** (Figs 1B, 4B). Total length 18.14. Carapace 6.72 long, 6.33 wide; abdomen 11.62 long, 7.71 wide. Carapace yellow-brown, margin gray-brown, with radiating dark-brown radial furrow. Dorsum of abdomen dark brown, with sagittal cardiac pattern and four symmetrical muscular impressions on either side. Eye sizes and interdistances: AME 0.35, ALE 0.31, PME 0.35, PLE 0.40; AME–AME 0.28, AME–ALE 0.16, PME–PME 0.24, PME–PLE 0.47. Clypeus height 0.44. Chelicerae brown, with three promarginal and three retromarginal teeth. Spination of left leg I: Fe 3d 5p 5r; Pa 1d 1p 1r; Ti 2d 2p 2r 4-4v; Mt 3d 3p 3r 3-0v. Leg measurements: I 32.95 (8.72, 12.14, 8.87, 3.22); II 35.91 (9.62, 13.28, 9.73, 3.28); III 28.55 (7.93, 9.69, 8.10, 2.83); IV 32.08 (8.64, 11.01, 9.26, 3.17).

***Epigyne*** (Fig. 3). Epigynal plate ca 1.3 times wider than long (as long as wide in holotype). Anterior part of median plate (MP) 1.5 times wider than posterior part (equal in holotype). Copulatory openings (CO) slit-like, located mid ventrally on between lateral walls (LW) and median plate. Copulatory ducts (CD) wrapped 3 times around base of spermatheca (BS); spermatheca head (SH) almost spherical (elongated oval in holotype). Fertilization ducts (FD) crescent-shaped.

##### Distribution.

**Figure 7.**
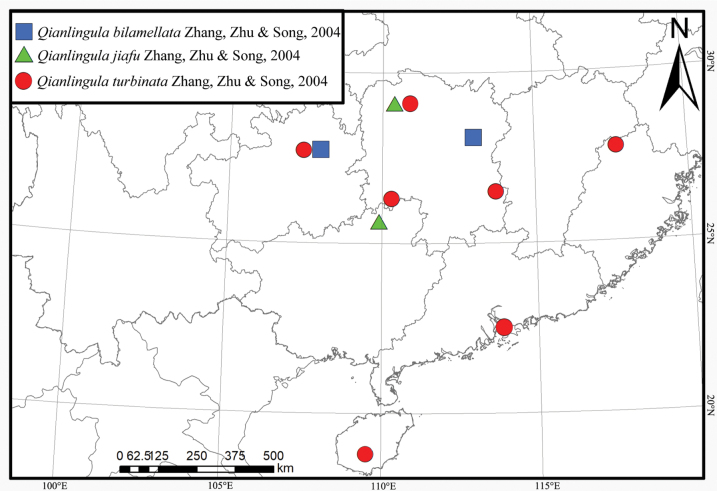
Distribution of *Q. bilamellata*, *Q. jiafu* and *Q. turbinata* in China.

China (Hunan, Guangxi) (Yin et al. 2012) (Fig. 7).

#### ﻿ Qianlingula turbinata

Taxon classificationAnimaliaAraneaePisauridae

7977990D-361A-565D-B04C-9A68B40B034C

Figs 1C–D, 5, 6, 7

Qianlingula turbinata Zhang et al., 2004: 402, figs 160–162 (♀); Yin et al. 2012: 896, fig. 451a–c (♀).

##### Material examined.

***Holotype*** • ♀, China, Hunan, Chengbu Co., 20.08.1982, J.F. Wang leg. (MHBU, Figs 6A, C). ***Paratypes*** • 1♂1♀, China, Hainan, Wuzhishan Ct., Emerald Park, Taiping Cr., 18°47'34"N, 109°31'33"E, elev. 381 m, 17.02.2024, Q.L. Lu et al. leg. (SWUC, Figs 5, 6B, D).

##### Diagnosis.

This species resembles *Q. jiafu* (Figs 2–4) in having bifurcated retrolateral tibial apophysis (RTA), long and slender embolus (E), spine-like embolic process of male palp (EBP), slit-like copulatory openings (CO) of epigyne, but differs by larger body size (27.45–34.33) (Fig. 1C, D, vs 16.23–18.14); margin of anterior branch of retrolateral tibial apophysis more curled (Fig. 5E–H, vs thick, Fig. 2E–H); cymbial furrow (CF) 2 times shorter than cymbium (Fig. 5E, vs 0.6 times of cymbium length, Fig. 2E); tip of cymbium longer than bulb (Fig. 5E, vs shorter, Fig. 2E); tip of conductor (C) blunt in ventral view (Fig. 5B, vs pointed, Fig. 2B). Posterior part of median plate of epigyne narrower than half width of anterior part (Fig. 6A, B, vs wider, Fig. 3A, B).

##### Description.

**Male** (Fig. 1C). Total length 27.45. Carapace 13.91 long, 11.94 wide; abdomen 13.54 long, 8.89 wide. Carapace yellow-brown, margin dark brown, with eight red-brown radiating furrows. Dorsum of abdomen brown, cardiac pattern rhomboid yellow-brown with pair dark-brown muscular impression, with heart-shaped depression, two white spots at heart angles. Eye sizes and interdistances: AME 0.65, ALE 0.51, PME 0.62, PLE 0.69; AME–AME 0.31, AME–ALE 0.26, PME–PME 0.43, PME–PLE 0.77. Clypeus height 1.33. Spination of left leg I: Fe 3d 5p 5r; Pa 1d 1p 1r; Ti 2d 2p 2r 4-4v; Mt 3d 3p 3r 3-0v. Leg measurements: I 76.04 (21.00, 27.95, 20.67, 6.42); II 84.46 (23.03, 31.05, 23.45, 6.93); III 76.65 (21.05, 27.51, 21.58, 6.51); IV 85.64 (22.16, 29.95, 25.77, 7.76).

***Palp*** (Fig. 5). Tibia ca 2 times longer than wide in retrolateral view, ca 0.4 of femur length, as long as patella, with two strong prolateral spines; tibial apophysis bifurcated, anterior branch with lamellar and curled margin, posterior branch claw-like. Cymbium droplet-shaped, 2.8 time longer than wide; cymbial furrow (CF) as long as half of cymbium; tip longer than bulb. Bulb elliptical; proximal terminal part of tegulum (T) curved; subtegulum (St) triangular in retrolateral view; median apophysis (MA) located centrally, hook-like, curved ventrally. Conductor (C) crescent-shaped in ventral view, with groove (holding fulcrum (Ful) and embolus (E)), with pointed tip. Embolus complex: with fulcrum (Ful) and basal process (EBP); fulcrum curved clockwise as long as embolus, with groove anteriorly; basal process spine-like, slightly curved at posterior end. Embolus proper originating at approximately 6-o’clock position, long, running clockwise surrounded tegulum, distal part resting in long groove of fulcrum.

**Female** (Fig. 1D). Total length 34.33. Carapace 15.08 long, 12.75 wide; abdomen 19.0 long, 12.56 wide. Carapace yellow-brown, margin dark brown, with red-brown radiating furrows. Fovea longitudinal, dark brown. Dorsum of abdomen dark brown, cardiac pattern rhomboid yellow-brown with pair of brown muscular impressions, with heart-shaped depression, two white spots at heart angles. Eye sizes and interdistances: AME 0.70, ALE 0.59, PME 0.70, PLE 0.85; AME–AME 0.32, AME–ALE 0.34, PME–PME 0.37, PME–PLE 0.78. Clypeus height 1.56. Chelicerae brown, with three promarginal and three retromarginal teeth. Spination of left leg I: Fe 3d 5p 5r; Pa 1d 1p 1r; Ti 2d 2p 2r 4-4v; Mt 3d 3p 3r 3-0v. Leg measurements: I 74.91 (20.17, 27.85, 20.35, 6.54); II 83.29 (23.29, 30.25, 22.56, 7.19); III 75.09 (21.30, 25.82, 21.06, 6.91); IV 86.10 (23.52, 29.15, 25.60, 7.83).

***Epigyne*** (Fig. 6). Epigynal plate pentagonal, almost as wide as long. Anterior part of median plate 2 times wider than posterior part, almost Y-shaped. Copulatory openings (CO) slit-like, located mid ventrally on between lateral walls (LW) and median plate (MP). Copulatory ducts (CD) wrapped 3 times around base of spermatheca (BS), forming round figure; first loop of CD touching each other, and other loops slightly spaced; spermatheca head (SH) almost heart-shaped. Fertilization ducts (FD) crescent-shaped.

##### Distribution.

China (Hunan, Fujian, Guizhou, Guangdong and Hainan) (Yin et al. 2012; Zhang and Wang 2017) (Fig. 7).

##### Remarks.

Yin et al. (2012) recorded this species from Hunan, Fujian, and Hainan provinces in China. Zhang and Wang (2017) recorded it from Guangdong, with a photo of female habitus. So, this is a widely distributed species reported in four provinces in Southern China.

Both *Q. jiafu* and *Q. turbinata* inhabit rocky shoals along streams, hiding under rocks when sensing danger or during the day. *Qianlingula jiafu* prefers slower-moving waters, while *Q. turbinata* thrives in fast-flowing currents. Notably, both species possess dense bristles on their walking legs—particularly on the tibia, metatarsus, and tarsus—which likely facilitate rapid movement across the water surface, an adaptation to their semi-aquatic hunting behavior. This morphological specialization warrants further investigation.

## Supplementary Material

XML Treatment for Qianlingula

XML Treatment for Qianlingula jiafu

XML Treatment for Qianlingula turbinata

## ﻿Additional information

### Use of AI

No use of AI was reported.

### Author ORCIDs

Tian-Yu Ren https://orcid.org/0000-0002-3680-6060

Qian-Le Lu https://orcid.org/0000-0002-5250-3473

Zhi-Sheng Zhang https://orcid.org/0000-0002-9304-1789

### Data availability

All of the data that support the findings of this study are available in the main text.
